# Privacy-preserving synthetic data from a national CIDP registry: validation against real-world evidence

**DOI:** 10.3389/fneur.2026.1839538

**Published:** 2026-10-13

**Authors:** Alessio Signori, Erika Schirinzi, Fiore Manganelli, Dario Cocito, Yuri Matteo Falzone, Chiara Briani, Anna Mazzeo, Angelo Schenone, Vincenzo Di Stefano, Giuseppe Cosentino, Girolama Alessandra Marfia, Luana Benedetti, Marinella Carpo, Massimiliano Filosto, Luca Leonardi, Marco Luigetti, Sabrina Matà, Giuseppe Piscosquito, Tiziana Rosso, Marta Lucchetta, Gabriele Siciliano, Giuseppe Lauria Pinter, Maurizio Inghilleri, Teresa Anna Cantisani, Francesca Notturno, Dario Ricciardi, Francesco Habetswallner, Elisa Vegezzi, Federica Ginanneschi, Antonio Todisco, Roberta Brusa, Stefano Sotgiu, Paolo Solla, Alberto De Lorenzo, Claudia Lozi, Alessandro Salvalaggio, Adele Ratti, Luca Gentile, Giorgia Mataluni, Filippo Brighina, Barbara Risi, Loris Poli, Francesca Forcina, Francesca Vitali, Federica Moret, Fabrizio Canale, Emanuele Cassano, Eleonora Dalla Bella, Stefania Casali, Vincenzo Di Lazzaro, Alessandro Cesare Prelle, Alessandra Carta, Filippo De Mattei, Giuseppe De Biasi, Eduardo Nobile-Orazio, Pietro Emiliano Doneddu

**Affiliations:** 1 Department of Health Sciences, University of Genova, Genova, Italy; 2 Department of Clinical and Experimental Medicine, Neurological Clinic, University of Pisa, Pisa, Italy; 3 Department of Neurosciences, Reproductive and Odontostomatological Sciences, University of Naples Federico II, Naples, Italy; 4 Dipartimento Scienze Cliniche e Biologiche, Università di Torino, Turin, Italy; 5 Neurology Unit, IRCCS San Raffaele Scientific Institute, Milan, Italy; 6 Department of Neuroscience, University of Padova, Padova, Italy; 7 Unit of Neurology and Neuromuscular Diseases, Department of Clinical and Experimental Medicine, University of Messina, Messina, Italy; 8 Department of Neuroscience, Rehabilitation, Ophthalmology, Genetics, Maternal and Child Health (DiNOGMI), University of Genova, Genova, Italy; 9 IRCCS Ospedale Policlinico San Martino, Genova, Italy; 10 Department of Biomedicine, Neuroscience, and Advanced Diagnostic (BIND), University of Palermo, Palermo, Italy; 11 Department of Brain and Behavioral Sciences, University of Pavia, Pavia, Italy; 12 Translational Neurophysiology Research Section, IRCCS Mondino Foundation, Pavia, Italy; 13 Dysimmune Neuropathies Unit, Department of Systems Medicine, Tor Vergata University of Rome, Roma, Italy; 14 ASST Bergamo Ovest-Ospedale Treviglio, Treviglio, Italy; 15 Department of Clinical and Experimental Sciences, NeMO-Brescia Clinical Center for Neuromuscular Diseases, University of Brescia, Brescia, Italy; 16 Neuromuscular and Rare Disease Centre, Department of Neuroscience, Mental Health and Sensory Organs (NESMOS), SAPIENZA University of Rome, Sant'Andrea Hospital, Rome, Italy; 17 Department of Neurosciences, Università Cattolica del Sacro Cuore, Rome, Italy; 18 Fondazione Policlinico Universitario A. Gemelli IRCCS, UOC Neurologia, Rome, Italy; 19 Department of Neurological and Psychiatric Sciences, Azienda Ospedaliero-Universitaria di Careggi, Florence, Italy; 20 Neurology Unit, University Hospital "San Giovanni di Dio e Ruggi d'Aragona", Salerno, Italy; 21 UOC di Neurologia, Ospedale San Bassano, Vicenza, Italy; 22 UOC Neurologia, Ospedale Santa Maria della Misericordia, Rovigo, Italy; 23 Unit of Neuroalgology, IRCCS Foundation 'Carlo Besta' Neurological Institute, Milano, Italy; 24 Department of Medical Biotechnology and Translational Medicine, Milan University, Milano, Italy; 25 Department of Human Neuroscience, Sapienza University of Rome, Rome, Italy; 26 IRCCS Neuromed, Pozzilli, Italy; 27 Servizio di Neurofisiopatologia, Azienda Ospedaliera di Perugia, Perugia, Italy; 28 Servizio di Neurofisiopatologia, Ospedale SS di Pescara, Pescara, Italy; 29 Clinical Neurophysiology Unit, Cardarelli Hospital, Naples, Italy; 30 Neuroimmunology Laboratory and Neuroimmunology Research Section, IRCCS Mondino Foundation, Pavia, Italy; 31 Department of Medical, Surgical and Neurological Sciences, University of Siena, Siena, Italy; 32 Department of Medicine and Surgery, Unit of Neurology, Neurophysiology, Neurobiology and Psychiatry, Università Campus Bio-Medico di Roma, Roma, Italy; 33 Fondazione Policlinico Universitario Campus Bio-Medico, Roma, Italy; 34 Neurologia-Stroke Unit, Dipartimento Di Neuroscienze, ASST Ovest MI, Sede Di Legnano, Italy; 35 Division of Child Neuropsychiatry, Department of Medicine, Surgery and Pharmacy, University of Sassari, Sassari, Italy; 36 Neurology Unit, University Hospital of Sassari, Sassari, Italy; 37 Neuromuscular and Neuroimmunology Unit, IRCCS Humanitas Research Hospital, Milan, Italy; 38 Unit of Neurology, ASST Spedali Civili, Brescia, Italy; 39 Neurology and Stroke Unit, Ospedale del Mare, ASL Napoli 1 Centro, Napoli, Italy; 40 Neurology Unit Patologie Neurologiche e Specialistiche, Azienda Ospedaliero-Universitaria San Luigi Gonzaga, Orbassano, Italy; 41 Department of Medicine, Surgery and Dentistry, Center of Neurodegenerative Diseases (CEMAND), University of Salerno, Fisciano, Italy; 42 Department of Biomedical Sciences, Humanitas University, Milan, Italy

**Keywords:** artificial intelligence, chronic inflammatory demyelinating polyradiculoneuropathy, CIDP, real-world data, synthetic data, epidemiology, risk factors

## Abstract

**Background:**

Research in chronic inflammatory demyelinating polyradiculoneuropathy (CIDP) is constrained by low prevalence, limited data sharing, and privacy regulations. Disease registries mitigate some of these barriers, yet the reuse of individual-level data remains challenging. Artificial intelligence– and regression-based synthetic data (SD) generation may offer a privacy-preserving solution, provided analytical validity is maintained. We aimed to validate SD derived from the Italian CIDP Registry using a published case–control study as a reference framework.

**Methods:**

We analyzed a matched case–control dataset including 195 CIDP cases and 195 controls to examine lifestyle and dietary factors associated with CIDP risk. SD were generated using sequential conditional models stratified by sex. Fidelity was assessed by comparing real and SD in terms of marginal distributions, data distinguishability (propensity score mean squared error), and preservation of associations with CIDP risk using univariable and multivariable logistic regression.

**Results:**

SD preserved the original case–control balance and sex distribution. Marginal distributions showed high concordance between real and SD, with minor smoothing in a small number of multi-level dietary variables. Distinguishability analyses indicated low separability between real and synthetic records. Univariable associations with CIDP risk were closely replicated, including the protective effects of higher fish and rice intake. Multivariable models demonstrated highly concordant effect estimates between datasets.

**Conclusion:**

SD derived from the Italian CIDP Registry accurately reproduces key analytical properties of real-world data while preserving patient privacy. This approach enables reproducible and shareable CIDP research and may support future applications, including synthetic standard-of-care comparator cohorts and precision medicine–oriented investigations.

## 1 Introduction

Chronic inflammatory demyelinating polyradiculoneuropathy (CIDP) is a rare but treatable immune-mediated neuropathy characterized by marked clinical, immunological, and treatment-response heterogeneity (1). Both observational research and clinical trials in CIDP are intrinsically constrained by low disease prevalence, which hampers patient enrollment and limits the assembly of cohorts sufficiently large and representative to capture the full spectrum of disease variability and support robust, generalizable inferences (2–5). Over time, these structural constraints have translated into prolonged recruitment periods, suboptimal enrollment, selection bias, and high costs for interventional studies, while observational research has frequently relied on relatively small case series (3–11). As a result, investigations into environmental determinants and genetic susceptibility in CIDP remain limited in number and scope, restricting our ability to comprehensively characterize disease risk architecture and biological heterogeneity (12–17).

To address some of these challenges, disease-specific registries have been established in recent years to collect real-world data from large, multicenter patient populations (12, 13, 18, 19). Within this landscape, the Italian CIDP Registry has contributed to CIDP research by supporting epidemiological investigations, observational studies, and clinical trials in this rare disease (5, 12, 13). Real-world datasets, however, are frequently affected by missing data, heterogeneity in data collection, and internal inconsistencies. In addition, data sharing across registries and with external investigators remains heavily constrained by privacy and regulatory requirements, limiting reproducibility, independent validation, and secondary analyses.

Generative artificial intelligence (AI) and parametric and semi-parametric regression-based generative models offer a potential solution to these challenges through the generation of synthetic data (SD), which are trained to learn and reproduce the key statistical features of real-world datasets while removing the risk of patient re-identification (20–26). Applications of SD across several biomedical research areas, including hematology, oncology, and neurology, have shown that synthetic datasets can preserve high fidelity to original data distributions, maintain clinically meaningful associations and treatment-effect estimates, and support privacy-preserving, reproducible research (20–26). These characteristics are particularly relevant in rare diseases such as CIDP, where limited sample sizes and restricted data sharing represent major barriers to robust evidence generation.

Within this evolving methodological framework, the Italian CIDP registry offers an opportunity to evaluate the validity of SD generation in this rare disease setting. Such validation requires a previously conducted, well-characterized analysis in which data structure, analytical strategy, and clinical interpretation are firmly established. Among the studies performed within the Italian CIDP registry, a multicenter case–control investigation examined the role of lifestyle and dietary factors in CIDP risk (12). This study provides a rigorously curated dataset with clearly defined variables, outcomes, and analytical assumptions, making it particularly well-suited as a reference framework to assess whether generated SD can faithfully reproduce real-world, registry-based research.

The aim of the present study was to validate regression-based SD generated from the Italian CIDP Registry using a previously published case–control investigation on lifestyle and dietary factors as a reference framework. Specifically, we assessed whether a fully synthetic, non-identifiable dataset could faithfully reproduce (i) the marginal distributions of dietary and lifestyle variables, (ii) the univariable associations with CIDP risk, and (iii) the multivariable joint structure of the key dietary predictors identified in the original analysis.

## 2 Methods

### 2.1 Reference study: data source and study population

The present validation study is based on a previously described multicenter epidemiological case–control investigation conducted within the Italian CIDP Registry (12). That study included patients diagnosed with CIDP according to the 2010 European Federation of Neurological Societies/Peripheral Nerve Society (EFNS/PNS) criteria, enrolled in a national web-based database (27). At enrollment, standardized clinical data were collected by the treating neurologist, including disease history and disability measures.

The original analysis examined the prevalence of lifestyle and dietary habits in patients with CIDP (12). Only patients who reported no change in lifestyle or dietary habits under investigation after disease onset were included. Healthy controls were identified among partners of patients with CIDP, and identical information was collected using the same assessment tools. Because patients and controls differed markedly by sex and were likely to share environmental habits, a 1:1 sex-matched case–control design with randomly selected controls was adopted.

Lifestyle and dietary exposures were assessed using a structured questionnaire administered identically to cases and controls. Items included exposure to toxic agents (prolonged versus never/occasional), smoking (including duration and amount of exposure), illicit drug use (repeated versus never/occasional), alcohol consumption (including amount of exposure), dietary regimen (vegan, vegetarian, macrobiotic, omnivorous, or other), and frequency of consumption of a variety of foods (one or more times per day, three to four times per week, one to two times per week, two to three times per month). Food items included pasta, rice, meat, raw meat, white meat, fish, vegetables, fruit, cheese, eggs, sweets, coffee, tea, milk, and soft drinks.

Information on antecedent events occurring within 1–42 days before symptom onset was also collected in the original study; however, these data were not the primary focus of the present SD validation and are not further addressed here.

All data were entered into a centralized web-based platform developed by CINECA (Bologna, Italy).

In the original study, associations between lifestyle and dietary variables and CIDP risk were evaluated using logistic regression models with case–control status as the dependent variable. Each exposure was analyzed separately, and odds ratios (OR) with corresponding confidence intervals (CI) were estimated. Statistical significance was set at a two-sided *p-value* <0.05.

### 2.2 Statistical methods

For the present validation study, we analyzed the matched case–control dataset of 390 individuals (195 CIDP cases and 195 controls) from the original investigation. To ensure comparability with the published analyses, categorical variables derived from the original questionnaire were harmonized to match the exposure levels reported in the reference tables. For dietary variables, frequency categories were collapsed as “Never,” “<1,” “≤2,” “1–2,” “≥3,” “3–4,” “≥5” times per week, while binary variables were coded as “Yes/No.” Missing or not-available categories were retained for descriptive analyses but excluded from regression models and goodness-of-fit procedures sensitive to sparse cells.

Sex was treated as a design variable rather than as an outcome or predictor. To generate privacy-preserving data, we used the synthpop package in R and created synthetic datasets stratified by sex, thereby preserving the original 1:1 matching scheme and stabilizing model estimation within each stratum (28). Within each stratum, predictor variables were synthesized first and the endpoint variable (case vs. control) last, using logistic regression for binary variables and multinomial regression for multi-level categorical variables. The sex-specific synthetic datasets were subsequently combined, and the case–control ratio (195:195) was verified in the final synthetic dataset.

Fidelity of the SD was evaluated using complementary validation approaches. First, marginal distributions of categorical variables in the real and synthetic datasets were compared using information-theoretic distance measures, including Total Variation (TV), Jensen–Shannon (JS), and Hellinger distances, with values closer to zero indicating better agreement. Classical χ^2^ goodness-of-fit tests were also performed, and Cramér’s V was reported as a sample-size–robust measure of effect size.

Privacy preservation was assessed using both record-level disclosure risk metrics and distinguishability-based measures. Record-level risk was evaluated by computing the exact match rate between synthetic and real records and further examined using nearest-neighbor proximity measures. Specifically, for categorical variables, the minimum Hamming distance between each synthetic record and its closest real counterpart was computed, with smaller distances indicating higher similarity and potential disclosure risk.

Distinguishability between real and synthetic records was further assessed using the propensity score mean squared error (pMSE). A combined dataset labeled by data source (real vs. synthetic) was constructed, and a logistic classifier including all predictors as factors was fitted. Standardized pMSE (SpMSE) values were reported to facilitate comparison across model sizes. To avoid instability related to empty or sparse cells, pMSE analyses were performed on complete cases, consistent with the handling of sparse categories in other goodness-of-fit assessments. For all privacy metrics, lower values indicate reduced disclosure risk. Propensity score models were fitted using complete cases because logistic discrimination requires complete predictor information. Given the very low proportion of missing observations (≤1% for all variables), we considered the potential impact of complete-case analysis on pMSE estimates to be negligible.

To assess whether associations with the outcome were preserved, we fitted univariable logistic regression models with case–control status as the dependent variable in both the real and synthetic datasets, using identical reference categories. OR with 95% CI were estimated and compared, and the results were visualized using forest plots. In addition, descriptive bar plots were generated to compare category-level proportions between real and synthetic data, overall and stratified by case–control status, to facilitate visual inspection of potential systematic discrepancies.

Although the original study focused on univariable associations, we conducted an exploratory multivariable logistic regression to evaluate whether the synthetic dataset preserved the joint structure of key predictors. To minimize overfitting in the context of a modest sample size, we fitted a parsimonious model including only variables that showed stable and statistically significant associations (*p* < 0.05) in both real and synthetic univariable analyses. Variables that appeared significant only in the SD were deliberately excluded from multivariable modeling, as such findings would likely reflect properties of the generative process rather than new clinical evidence; these variables were instead examined descriptively and through univariable analyses.

All statistical analyses were performed using R version 4.5.1 (R Foundation for Statistical Computing). The fully synthetic, non-identifiable dataset generated for this study is provided as Supplementary Dataset S1.

## 3 Results

The matched analytic dataset comprised 390 individuals, including 195 CIDP cases and 195 controls. SD generation preserved both the case–control balance and the imposed sex distribution (204 women and 186 men), allowing direct comparison of descriptive summaries and association measures between real and synthetic datasets.

Marginal distributions of categorical predictors were closely replicated in the SD (Supplementary Tables 1, 2; Figure 1). The largest absolute differences were observed for a limited number of multi-level dietary variables, particularly meat (≥3 times/week: 6.15% difference among cases), white meat (<1 time/week: 5.64% difference among cases), and cheese (<1 time/week: −6.15% among cases). For the remaining variables, discrepancies were minimal.

**Figure 1:**
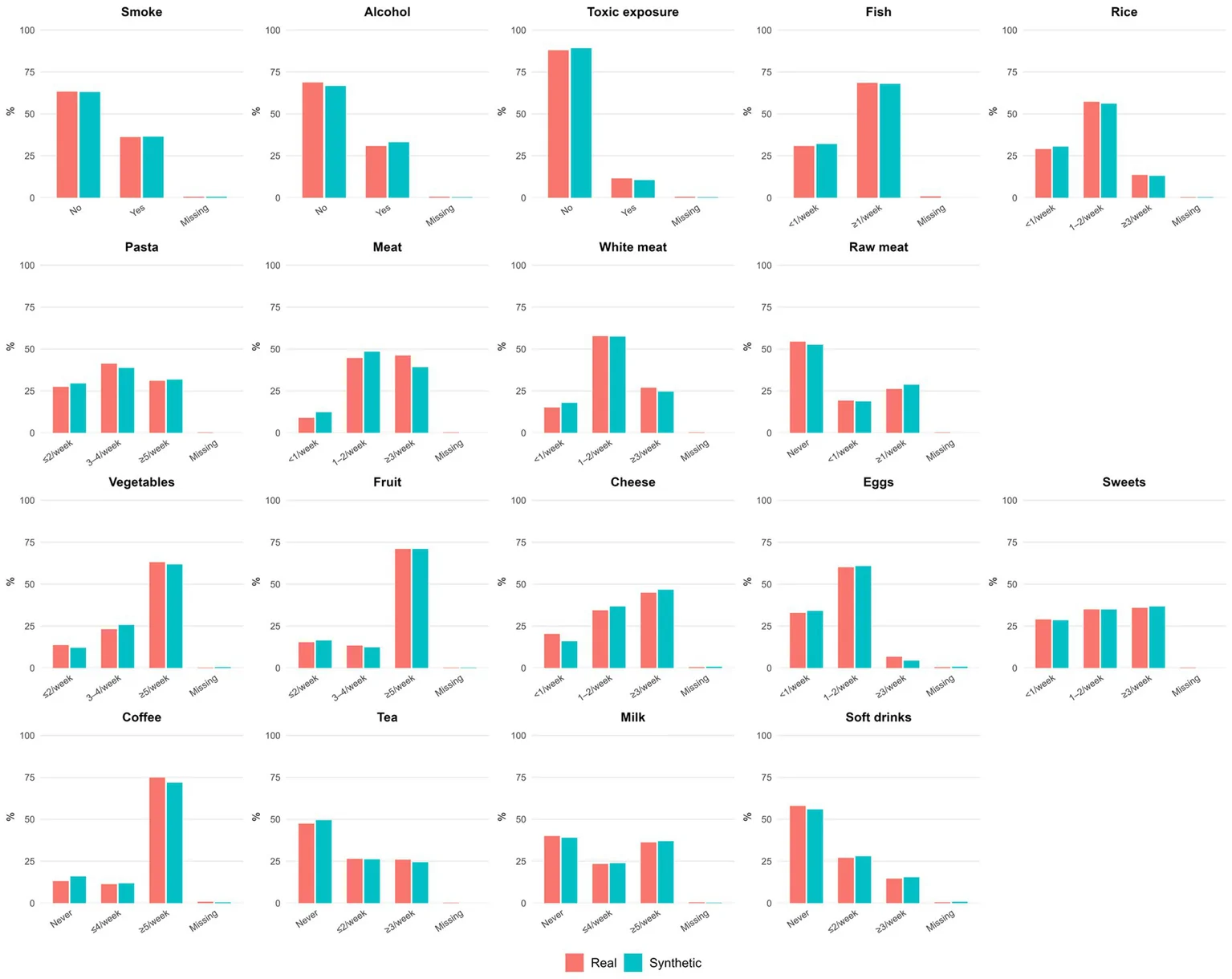
Marginal distributions of lifestyle and dietary variables in real and synthetic datasets. Bar plots show the proportion (%) of individuals in each exposure category among CIDP cases (upper panels) and controls (lower panels). Red bars indicate real data and blue bars indicate synthetic data. Variables include alcohol consumption, cheese, coffee, eggs, fish, fruit, meat, milk, pasta, raw meat, rice, smoking, soft drinks, sweets, tea, toxic exposure, vegetables, and white meat.

Consistent with these findings, information-theoretic distance measures indicated high agreement between real and SD. Overall fidelity metrics are presented in Table 1, whereas the corresponding analyses stratified by case–control status are reported in Supplementary Table 3. Total variation distances were small across variables, typically ranging from 0.01 to 0.05 (Table 1; Supplementary Table 3), while Jensen–Shannon and Hellinger distances were near zero. Classical goodness-of-fit tests supported these results, with generally high χ^2^ *p-values* and uniformly small Cramér’s V values (≤0.09), indicating negligible differences between real and synthetic distributions. The modest departures observed were confined to a few multi-level dietary variables and reflected mild smoothing of extreme frequencies rather than systematic distortion.

**Table 1:** Summary of analytical utility and privacy metrics comparing the real and synthetic datasets.

Predictor	TV	Hellinger	Cramér’s V	sPMSE
Meat	0.072	0.066	0.087	5.85
Cheese	0.044	0.042	0.059	2.68
Coffee	0.033	0.032	0.045	1.57
White meat	0.028	0.046	0.055	2.34
Pasta	0.028	0.041	0.046	1.65
Vegetables	0.028	0.029	0.04	1.27
Raw meat	0.026	0.041	0.045	1.61
Alcohol	0.023	0.023	0.032	0.78
Eggs	0.023	0.038	0.053	2.2
Soft drinks	0.021	0.018	0.025	0.5
Tea	0.021	0.039	0.042	1.36
Rice	0.015	0.012	0.017	0.23
Toxic exposure	0.013	0.019	0.027	0.56
Milk	0.013	0.017	0.024	0.44
Fish	0.013	0.063	0.063	3.11
Fruit	0.01	0.014	0.019	0.29
Sweets	0.008	0.036	0.037	1.05
Smoking	0.003	0.002	0.003	0.01
Gender	0	0	0	0

Propensity score–based analyses further corroborated the fidelity of the SD. The global pMSE was approximately 0.010 (standardized pMSE: 31.9; 52 degrees of freedom), which is acceptable given the number of predictors included in the model. Variable-specific standardized pMSE values were low to moderate and were mainly driven by multi-level dietary items (meat, fish, white meat, and cheese), whereas most other variables showed values ≤1.7, and smoking showed no distinguishability. No identical matches were observed between synthetic and original records (exact match rate = 0%). Nearest-neighbor disclosure risk analysis based on Hamming distance indicated that synthetic records were generally well separated from real individuals. The minimum distance to the closest real record had a median of 5 (IQR: 4–5; range: 1–8), and only 0.51% (2/390 records) of synthetic records exhibited a distance ≤1, indicating a very low prevalence of near-duplicate records. Consistently, additional disclosure risk metrics, including the pMSE, indicated low distinguishability between real and SD while maintaining high analytical fidelity.

Univariable logistic regression analyses demonstrated close agreement between real and synthetic datasets in both the direction and magnitude of associations with CIDP risk (Figure 2; Supplementary Table 4). The protective associations observed in the original study for fish intake (≥1 time/week vs. < 1 time/week) and rice consumption were preserved in the SD, with OR below unity and overlapping CI. Smoking showed no association with CIDP risk in the real data and remained null in the synthetic dataset. For the remaining dietary and lifestyle variables, including pasta, vegetables, fruit, tea, coffee, milk, and soft drinks, OR and CI were highly concordant, with no evidence of systematic over- or underestimation. Where minor discrepancies were present, particularly for raw and white meat, they mirrored the modest marginal smoothing identified in the distributional analyses and did not alter the direction of effects.

**Figure 2:**
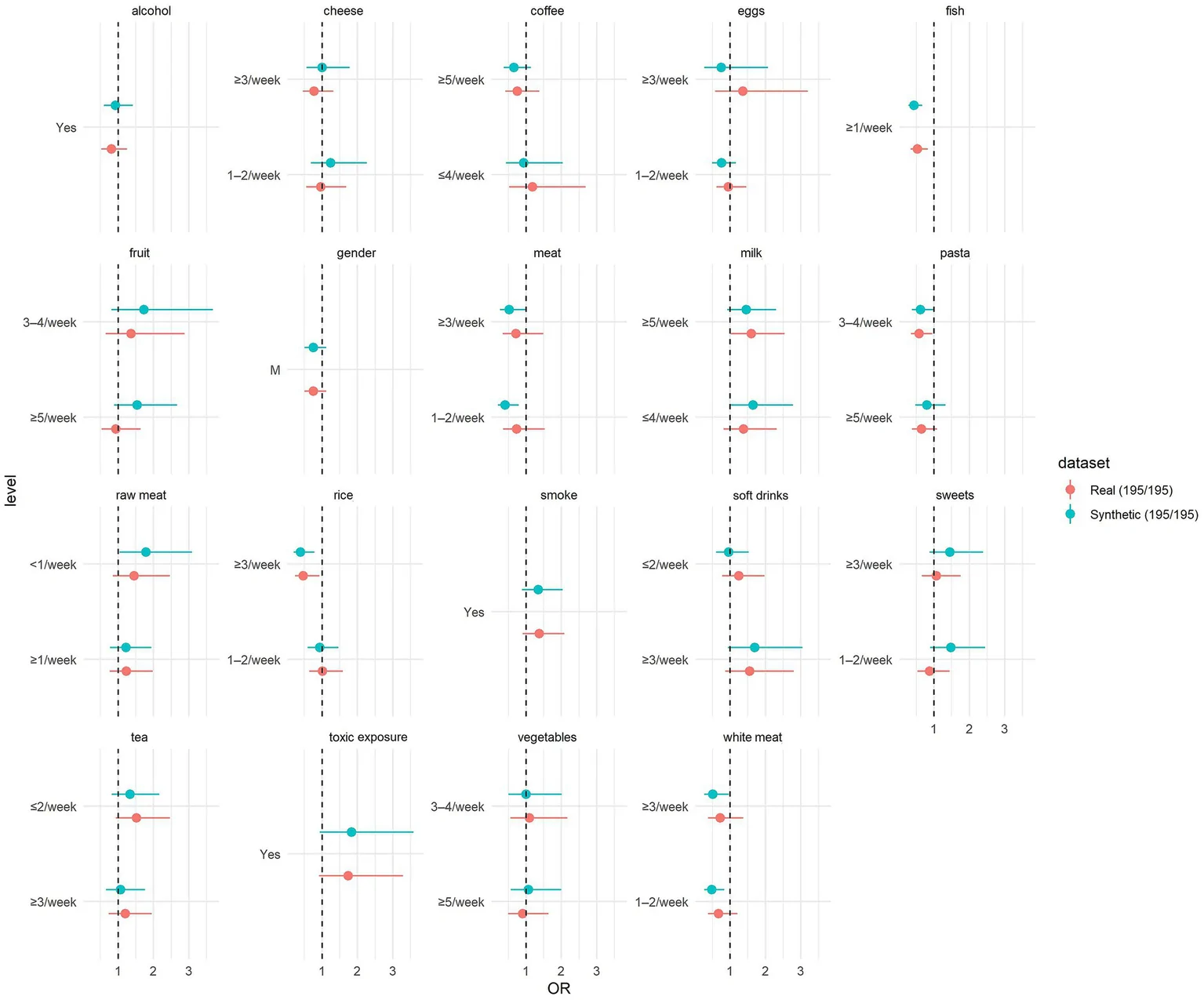
Univariable associations between lifestyle and dietary variables and CIDP risk in real and synthetic datasets. Forest plot comparing OR (circles) and 95% CI (horizontal lines) from univariable logistic regression models in real and synthetic datasets. Red circles represent estimates from real data and blue circles represent estimates from synthetic data. The vertical dashed line indicates an OR of 1. Reference category for categorical dietary variables is <1/week.

To evaluate whether the synthetic dataset preserved the joint structure of the primary predictors, we fitted identical multivariable logistic regression models in the real and SD, including only fish intake and rice intake, the two variables consistently associated with case–control status in univariable analyses. The resulting estimates showed very close agreement between datasets (Table 2). Higher fish consumption (≥1 time/week) remained protective in both analyses (real data: OR 0.52, 95% CI 0.33–0.81; synthetic data: OR 0.44, 95% CI 0.28–0.68). Similarly, higher rice intake (≥3 times/week) demonstrated comparable protective associations (real data: OR 0.45, 95% CI 0.24–0.85; SD: OR 0.43, 95% CI 0.22–0.84).

**Table 2:** Multivariable logistic regression analysis (real vs. synthetic datasets).

Predictor	Level (vs <1/week)	Real			Synthetic		
		OR	95% CI	*p*-value	OR	95% CI	*p*-value
Fish	≥1/week	0.52	0.33–0.81	0.004	0.44	0.28–0.68	<0.001
Rice	1–2/week	0.88	0.55–1.41	0.60	1.12	0.71–1.77	0.63
	≥3/week	0.45	0.24–0.86	0.015	0.43	0.22–0.84	0.013

## 4 Discussion

In this study, we demonstrate that regression-based SD generated from the Italian CIDP registry can faithfully reproduce the structure and key analytical properties of a previously conducted matched case–control study. The synthetic dataset preserved marginal distributions, univariable associations, and the joint multivariable structure of the primary predictors, with only minor and localized smoothing observed for a limited number of multi-level dietary variables. Importantly, statistically meaningful signals identified in the original analysis, such as the protective association of higher fish and rice intake, were retained in the SD with comparable effect sizes and CI. Together, these findings support the use of SD as a valid, privacy-preserving surrogate for real-world registry data in CIDP.

Beyond demonstrating analytical fidelity, this study also provides a fully synthetic patient-level dataset together with a comprehensive utility-privacy validation framework. By making these resources available as Supplementary material, we aim to facilitate reproducibility, independent methodological development, educational activities, and future collaborative research without requiring access to identifiable patient data. The replication of the protective associations for fish and rice intake warrants brief consideration. These findings are consistent with those reported in the original epidemiological investigation, where potential biological explanations are discussed in detail (12). Rice- and fish-derived bioactive compounds have been associated with antioxidant, anti-inflammatory, and immunomodulatory effects in experimental models, and higher fish consumption has been associated with a reduced risk of several autoimmune diseases (29–36). The present study was not designed to investigate causal mechanisms. Their preservation in the synthetic dataset should therefore be interpreted primarily as evidence that the generative model retained clinically meaningful statistical relationships rather than as confirmation of biological hypotheses. Nevertheless, the relevance of these observations to CIDP remains uncertain, and the observed associations should be interpreted cautiously and confirmed in independent studies. Importantly, their preservation in the SD indicates that the generative approach can retain subtle but clinically meaningful associations without distortion.

From a methodological perspective, SD offers several advantages for research in rare diseases. Registries such as the Italian CIDP Registry represent invaluable resources, yet their use is often constrained by privacy regulations, governance requirements, and the difficulties in sharing individual-level data across institutions. SD provides a practical solution by enabling data sharing without exposing identifiable patient information, thereby facilitating independent validation, secondary analyses, and methodological research. Similar approaches have been successfully applied in other biomedical settings, including large-scale multiple sclerosis registries, supporting the broader applicability of this strategy (20–26).

Beyond data sharing, SD may enable additional research applications that are particularly relevant for CIDP and other rare inflammatory neuropathies. CIDP is characterized by marked clinical heterogeneity, encompassing multiple phenotypic variants, diverse immunological mechanisms, and variable responses to immunomodulatory treatments (6–11). Consequently, many clinically meaningful subgroups are represented by small numbers even within large national registries. When appropriately validated, SD may support data augmentation for underrepresented subgroups, improving the feasibility of exploratory analyses and hypothesis generation in settings where sample size is a major limitation (20–26). Although these applications remain to be formally validated, synthetic datasets could facilitate exploratory work on patient stratification, diagnostic modeling (37) and hypothesis generation while preserving patient privacy. Synthetic datasets derived from high-quality registries could be used to train and evaluate diagnostic models and to assess the robustness of diagnostic features across disease variants, while minimizing privacy-related barriers to data reuse (38).

One promising future application of validated synthetic datasets is the development of synthetic standard-of-care comparator cohorts (39, 40). In CIDP, where randomized controlled trials may be difficult, costly, or ethically constrained once effective treatments are available, synthetic standard-of-care arms derived from registry data could support feasibility assessments, benchmarking, and hybrid trial designs. Such approaches have the potential to reduce patient burden and accelerate therapeutic development, provided that SD are generated and validated using robust methodological frameworks (39, 40).

Several limitations should be acknowledged. SD are inherently dependent on the quality, completeness, and structure of the source dataset and cannot generate information beyond what is contained in the original data. As observed here, variables with sparse categories may be subject to mild smoothing, which could affect the representation of extreme or very low-frequency groups. Importantly, this limitation does not preclude the use of SD to support analyses of underrepresented but systematically captured subgroups, which represent one of the main potential applications of this approach. However, investigations focusing on ultra-rare phenotypes or on very subtle biological or clinical correlations should preferentially rely on original patient-level data or on synthetic datasets generated using models specifically optimized for rare-event preservation. For these reasons, SD should be viewed as complementary tools that enhance accessibility and reproducibility, rather than as substitutes for original patient-level data in confirmatory or regulatory contexts. Accordingly, the principal value of the present synthetic dataset lies in supporting reproducibility, exploratory analyses, methodological development, and educational use, whereas confirmatory clinical inference should continue to rely on the original patient-level data. In addition, the present validation was conducted within the framework of a single matched case–control study with a binary outcome. Therefore, the generalizability of these findings to longitudinal designs, non-binary outcomes, time-to-event analyses, or more complex clinical endpoints cannot be assumed and warrants dedicated validation studies. Furthermore, the present study evaluated a single regression-based synthesis strategy. Future work directly comparing alternative generative approaches, including deep-learning architectures such as GANs and VAEs, will be important to determine whether similar utility and privacy characteristics can be achieved in neurological registry data.

Finally, pMSE was estimated using complete-case analysis. Although missingness was minimal in the present dataset, we acknowledge that sensitivity analyses under alternative missing-data assumptions would further strengthen future validations.

In conclusion, our findings indicate that SD can reliably support registry-based research in CIDP by preserving key analytical properties while mitigating privacy concerns. This approach may facilitate broader data sharing, enable novel research applications, and strengthen collaborative efforts in CIDP and other rare immune-mediated neuropathies, contributing to more efficient and sustainable research infrastructures. By increasing the scientific usability and value of real-world registry data, the implementation of validated SD generation may ultimately contribute to future precision medicine research in CIDP after validation in larger and more complex datasets.

## Data Availability

The fully synthetic, non-identifiable dataset generated for this study is provided as Supplementary Dataset S1. The anonymized real-world data used to generate the synthetic dataset are available from the corresponding author upon reasonable request, subject to applicable governance and privacy requirements.
