# Supplementary material for: Privacy-preserving synthetic data from a national CIDP registry: validation against real-world evidence
Source: Front Neurol. 2026 Oct 13;17:1839538. doi: 10.3389/fneur.2026.1839538 (PMC13710087; doi:10.3389/fneur.2026.1839538)
Supplement: Supplementary file 1 [file Table_1.DOCX]

**Supplementary Material**

**Supplementary Dataset S1.**
The excel file uploaded together the manuscript contains the fully synthetic patient-level dataset generated from the original matched case–control study (n = 390). All variables included in the analyses are provided using the same variable names, coding, and category definitions as the original dataset. The dataset is intended to support reproducibility, methodological development, educational use, and exploratory analyses without exposing identifiable patient information.

**Supplemental table 1** – Frequencies of each included variable in the original and synthetized database.

| **predictor** | **Level** | **Real** | | **Synthetic** | | **Diff (%)** |
| --- | --- | --- | --- | --- | --- | --- |
|  |  | **n** | **%** | **n** | **%** |  |
| Alcohol | No | 268 | 68.72 | 260 | 66.67 | -2.05 |
|  | Yes | 120 | 30.77 | 129 | 33.08 | 2.31 |
|  | Missing | 2 | 0.51 | 1 | 0.26 | -0.26 |
| Cheese | <1/week | 79 | 20.26 | 62 | 15.9 | -4.36 |
|  | 1–2/week | 134 | 34.36 | 143 | 36.67 | 2.31 |
|  | ≥3/week | 175 | 44.87 | 182 | 46.67 | 1.79 |
|  | Missing | 2 | 0.51 | 3 | 0.77 | 0.26 |
| Coffee | Never | 51 | 13.08 | 62 | 15.9 | 2.82 |
|  | ≤4/week | 44 | 11.28 | 46 | 11.79 | 0.51 |
|  | ≥5/week | 292 | 74.87 | 280 | 71.79 | -3.08 |
|  | Missing | 3 | 0.77 | 2 | 0.51 | -0.26 |
| Eggs | <1/week | 128 | 32.82 | 133 | 34.1 | 1.28 |
|  | 1–2/week | 234 | 60 | 237 | 60.77 | 0.77 |
|  | ≥3/week | 26 | 6.67 | 17 | 4.36 | -2.31 |
|  | Missing | 2 | 0.51 | 3 | 0.77 | 0.26 |
| Fish | <1/week | 120 | 30.77 | 125 | 32.05 | 1.28 |
|  | ≥1/week | 267 | 68.46 | 265 | 67.95 | -0.51 |
|  | Missing | 3 | 0.77 | 0 | 0 | -0.77 |
| Fruit | ≤2/week | 60 | 15.38 | 64 | 16.41 | 1.03 |
|  | 3–4/week | 52 | 13.33 | 48 | 12.31 | -1.03 |
|  | ≥5/week | 277 | 71.03 | 277 | 71.03 | 0 |
|  | Missing | 1 | 0.26 | 1 | 0.26 | 0 |
| Meat | <1/week | 35 | 8.97 | 48 | 12.31 | 3.33 |
|  | 1–2/week | 174 | 44.62 | 189 | 48.46 | 3.85 |
|  | ≥3/week | 180 | 46.15 | 153 | 39.23 | -6.92 |
|  | Missing | 1 | 0.26 | 0 | 0 | -0.26 |
| Milk | Never | 156 | 40 | 152 | 38.97 | -1.03 |
|  | ≤4/week | 91 | 23.33 | 93 | 23.85 | 0.51 |
|  | ≥5/week | 141 | 36.15 | 144 | 36.92 | 0.77 |
|  | Missing | 2 | 0.51 | 1 | 0.26 | -0.26 |
| Pasta | ≤2/week | 107 | 27.44 | 115 | 29.49 | 2.05 |
|  | 3–4/week | 161 | 41.28 | 151 | 38.72 | -2.56 |
|  | ≥5/week | 121 | 31.03 | 124 | 31.79 | 0.77 |
|  | Missing | 1 | 0.26 | 0 | 0 | -0.26 |
| Raw meat | Never | 212 | 54.36 | 205 | 52.56 | -1.79 |
|  | <1/week | 75 | 19.23 | 73 | 18.72 | -0.51 |
|  | ≥1/week | 102 | 26.15 | 112 | 28.72 | 2.56 |
|  | Missing | 1 | 0.26 | 0 | 0 | -0.26 |
| Rice | <1/week | 113 | 28.97 | 119 | 30.51 | 1.54 |
|  | 1–2/week | 223 | 57.18 | 219 | 56.15 | -1.03 |
|  | ≥3/week | 53 | 13.59 | 51 | 13.08 | -0.51 |
|  | Missing | 1 | 0.26 | 1 | 0.26 | 0 |
| Smoke | No | 247 | 63.33 | 246 | 63.08 | -0.26 |
|  | Yes | 141 | 36.15 | 142 | 36.41 | 0.26 |
|  | Missing | 2 | 0.51 | 2 | 0.51 | 0 |
| Soft drinks | Never | 226 | 57.95 | 218 | 55.9 | -2.05 |
|  | ≤2/week | 105 | 26.92 | 109 | 27.95 | 1.03 |
|  | ≥3/week | 57 | 14.62 | 60 | 15.38 | 0.77 |
|  | Missing | 2 | 0.51 | 3 | 0.77 | 0.26 |
| Sweets | <1/week | 113 | 28.97 | 111 | 28.46 | -0.51 |
|  | 1–2/week | 136 | 34.87 | 136 | 34.87 | 0 |
|  | ≥3/week | 140 | 35.9 | 143 | 36.67 | 0.77 |
|  | Missing | 1 | 0.26 | 0 | 0 | -0.26 |
| Tea | Never | 185 | 47.44 | 193 | 49.49 | 2.05 |
|  | ≤2/week | 103 | 26.41 | 102 | 26.15 | -0.26 |
|  | ≥3/week | 101 | 25.9 | 95 | 24.36 | -1.54 |
|  | Missing | 1 | 0.26 | 0 | 0 | -0.26 |
| Toxic exposure | No | 343 | 87.95 | 348 | 89.23 | 1.28 |
|  | Yes | 45 | 11.54 | 41 | 10.51 | -1.03 |
|  | Missing | 2 | 0.51 | 1 | 0.26 | -0.26 |
| Vegetables | ≤2/week | 53 | 13.59 | 47 | 12.05 | -1.54 |
|  | 3–4/week | 90 | 23.08 | 100 | 25.64 | 2.56 |
|  | ≥5/week | 246 | 63.08 | 241 | 61.79 | -1.28 |
|  | Missing | 1 | 0.26 | 2 | 0.51 | 0.26 |
| White meat | <1/week | 59 | 15.13 | 70 | 17.95 | 2.82 |
|  | 1–2/week | 225 | 57.69 | 224 | 57.44 | -0.26 |
|  | ≥3/week | 105 | 26.92 | 96 | 24.62 | -2.31 |
|  | Missing | 1 | 0.26 | 0 | 0 | -0.26 |

| **Supplemental table 2** – Frequencies of each included variable in the original and synthetized database stratified for cases and controls. | | | | | | | | | | | | | |
| --- | --- | --- | --- | --- | --- | --- | --- | --- | --- | --- | --- | --- | --- |
|  |  | CONTROLS | | | | | | CASES | | | | | |
| **predictor** | **level** | **Real** | | **Synthetic** | | **Diff (%)** | **Real** | | | **Synthetic** | | **Diff (%)** |  |
|  |  | **n** | **%** | **n** | **%** |  | **n** | | **%** | **n** | **%** |  |  |
| Alcohol | No | 129.00 | 66.15 | 128.00 | 65.64 | -0.51 | 139.00 | | 71.28 | 132.00 | 67.69 | -3.59 |  |
|  | Yes | 64.00 | 32.82 | 66.00 | 33.85 | 1.03 | 56.00 | | 28.72 | 63.00 | 32.31 | 3.59 |  |
|  | Missing | 2.00 | 1.03 | 1.00 | 0.51 | -0.51 | 0.00 | | 0.00 | 0.00 | 0.00 | 0.00 |  |
| Cheese | <1/week | 37.00 | 18.97 | 32.00 | 16.41 | -2.56 | 42.00 | | 21.54 | 30.00 | 15.38 | -6.15 |  |
|  | 1–2/week | 64.00 | 32.82 | 66.00 | 33.85 | 1.03 | 70.00 | | 35.90 | 77.00 | 39.49 | 3.59 |  |
|  | ≥3/week | 93.00 | 47.69 | 94.00 | 48.21 | 0.51 | 82.00 | | 42.05 | 88.00 | 45.13 | 3.08 |  |
|  | Missing | 1.00 | 0.51 | 3.00 | 1.54 | 1.03 | 1.00 | | 0.51 | 0.00 | 0.00 | -0.51 |  |
| Coffee | Never | 23.00 | 11.79 | 26.00 | 13.33 | 1.54 | 28.00 | | 14.36 | 36.00 | 18.46 | 4.10 |  |
|  | ≤4/week | 18.00 | 9.23 | 20.00 | 10.26 | 1.03 | 26.00 | | 13.33 | 26.00 | 13.33 | 0.00 |  |
|  | ≥5/week | 152.00 | 77.95 | 147.00 | 75.38 | -2.56 | 140.00 | | 71.79 | 133.00 | 68.21 | -3.59 |  |
|  | Missing | 2.00 | 1.03 | 2.00 | 1.03 | 0.00 | 1.00 | | 0.51 | 0.00 | 0.00 | -0.51 |  |
| Eggs | <1/week | 64.00 | 32.82 | 61.00 | 31.28 | -1.54 | 64.00 | | 32.82 | 72.00 | 36.92 | 4.10 |  |
|  | 1–2/week | 120.00 | 61.54 | 125.00 | 64.10 | 2.56 | 114.00 | | 58.46 | 112.00 | 57.44 | -1.03 |  |
|  | ≥3/week | 11.00 | 5.64 | 9.00 | 4.62 | -1.03 | 15.00 | | 7.69 | 8.00 | 4.10 | -3.59 |  |
|  | Missing | 0.00 | 0.00 | 0.00 | 0.00 | 0.00 | 2.00 | | 1.03 | 3.00 | 1.54 | 0.51 |  |
| Fish | <1/week | 47.00 | 24.10 | 45.00 | 23.08 | -1.03 | 73.00 | | 37.44 | 80.00 | 41.03 | 3.59 |  |
|  | ≥1/week | 146.00 | 74.87 | 150.00 | 76.92 | 2.05 | 121.00 | | 62.05 | 115.00 | 58.97 | -3.08 |  |
|  | Missing | 2.00 | 1.03 | 0.00 | 0.00 | -1.03 | 1.00 | | 0.51 | 0.00 | 0.00 | -0.51 |  |
| Fruit | ≤2/week | 30.00 | 15.38 | 38.00 | 19.49 | 4.10 | 30.00 | | 15.38 | 26.00 | 13.33 | -2.05 |  |
|  | 3–4/week | 22.00 | 11.28 | 22.00 | 11.28 | 0.00 | 30.00 | | 15.38 | 26.00 | 13.33 | -2.05 |  |
|  | ≥5/week | 143.00 | 73.33 | 135.00 | 69.23 | -4.10 | 134.00 | | 68.72 | 142.00 | 72.82 | 4.10 |  |
|  | Missing | 0.00 | 0.00 | 0.00 | 0.00 | 0.00 | 1.00 | | 0.51 | 1.00 | 0.51 | 0.00 |  |
| Meat | <1/week | 15.00 | 7.69 | 16.00 | 8.21 | 0.51 | 20.00 | | 10.26 | 32.00 | 16.41 | 6.15 |  |
|  | 1–2/week | 88.00 | 45.13 | 104.00 | 53.33 | 8.21 | 86.00 | | 44.10 | 85.00 | 43.59 | -0.51 |  |
|  | ≥3/week | 92.00 | 47.18 | 75.00 | 38.46 | -8.72 | 88.00 | | 45.13 | 78.00 | 40.00 | -5.13 |  |
|  | Missing | 0.00 | 0.00 | 0.00 | 0.00 | 0.00 | 1.00 | | 0.51 | 0.00 | 0.00 | -0.51 |  |
| Milk | Never | 88.00 | 45.13 | 86.00 | 44.10 | -1.03 | 68.00 | | 34.87 | 66.00 | 33.85 | -1.03 |  |
|  | ≤4/week | 44.00 | 22.56 | 41.00 | 21.03 | -1.54 | 47.00 | | 24.10 | 52.00 | 26.67 | 2.56 |  |
|  | ≥5/week | 63.00 | 32.31 | 68.00 | 34.87 | 2.56 | 78.00 | | 40.00 | 76.00 | 38.97 | -1.03 |  |
|  | Missing | 0.00 | 0.00 | 0.00 | 0.00 | 0.00 | 2.00 | | 1.03 | 1.00 | 0.51 | -0.51 |  |
| Pasta | ≤2/week | 44.00 | 22.56 | 50.00 | 25.64 | 3.08 | 63.00 | | 32.31 | 65.00 | 33.33 | 1.03 |  |
|  | 3–4/week | 88.00 | 45.13 | 84.00 | 43.08 | -2.05 | 73.00 | | 37.44 | 67.00 | 34.36 | -3.08 |  |
|  | ≥5/week | 63.00 | 32.31 | 61.00 | 31.28 | -1.03 | 58.00 | | 29.74 | 63.00 | 32.31 | 2.56 |  |
|  | Missing | 0.00 | 0.00 | 0.00 | 0.00 | 0.00 | 1.00 | | 0.51 | 0.00 | 0.00 | -0.51 |  |
| Raw meat | Never | 113.00 | 57.95 | 111.00 | 56.92 | -1.03 | 99.00 | | 50.77 | 94.00 | 48.21 | -2.56 |  |
|  | <1/week | 33.00 | 16.92 | 29.00 | 14.87 | -2.05 | 42.00 | | 21.54 | 44.00 | 22.56 | 1.03 |  |
|  | ≥1/week | 49.00 | 25.13 | 55.00 | 28.21 | 3.08 | 53.00 | | 27.18 | 57.00 | 29.23 | 2.05 |  |
|  | Missing | 0.00 | 0.00 | 0.00 | 0.00 | 0.00 | 1.00 | | 0.51 | 0.00 | 0.00 | -0.51 |  |
| Rice | <1/week | 54.00 | 27.69 | 55.00 | 28.21 | 0.51 | 59.00 | | 30.26 | 64.00 | 32.82 | 2.56 |  |
|  | 1–2/week | 106.00 | 54.36 | 105.00 | 53.85 | -0.51 | 117.00 | | 60.00 | 114.00 | 58.46 | -1.54 |  |
|  | ≥3/week | 35.00 | 17.95 | 35.00 | 17.95 | 0.00 | 18.00 | | 9.23 | 16.00 | 8.21 | -1.03 |  |
|  | Missing | 0.00 | 0.00 | 0.00 | 0.00 | 0.00 | 1.00 | | 0.51 | 1.00 | 0.51 | 0.00 |  |
| Smoke | No | 130.00 | 66.67 | 129.00 | 66.15 | -0.51 | 117.00 | | 60.00 | 117.00 | 60.00 | 0.00 |  |
|  | Yes | 63.00 | 32.31 | 64.00 | 32.82 | 0.51 | 78.00 | | 40.00 | 78.00 | 40.00 | 0.00 |  |
|  | Missing | 2.00 | 1.03 | 2.00 | 1.03 | 0.00 | 0.00 | | 0.00 | 0.00 | 0.00 | 0.00 |  |
| Soft drinks | Never | 120.00 | 61.54 | 112.00 | 57.44 | -4.10 | 106.00 | | 54.36 | 106.00 | 54.36 | 0.00 |  |
|  | ≤2/week | 50.00 | 25.64 | 57.00 | 29.23 | 3.59 | 55.00 | | 28.21 | 52.00 | 26.67 | -1.54 |  |
|  | ≥3/week | 24.00 | 12.31 | 23.00 | 11.79 | -0.51 | 33.00 | | 16.92 | 37.00 | 18.97 | 2.05 |  |
|  | Missing | 1.00 | 0.51 | 3.00 | 1.54 | 1.03 | 1.00 | | 0.51 | 0.00 | 0.00 | -0.51 |  |
| Sweets | <1/week | 56.00 | 28.72 | 63.00 | 32.31 | 3.59 | 57.00 | | 29.23 | 48.00 | 24.62 | -4.62 |  |
|  | 1–2/week | 72.00 | 36.92 | 64.00 | 32.82 | -4.10 | 64.00 | | 32.82 | 72.00 | 36.92 | 4.10 |  |
|  | ≥3/week | 67.00 | 34.36 | 68.00 | 34.87 | 0.51 | 73.00 | | 37.44 | 75.00 | 38.46 | 1.03 |  |
|  | Missing | 0.00 | 0.00 | 0.00 | 0.00 | 0.00 | 1.00 | | 0.51 | 0.00 | 0.00 | -0.51 |  |
| Tea | Never | 100.00 | 51.28 | 101.00 | 51.79 | 0.51 | 85.00 | | 43.59 | 92.00 | 47.18 | 3.59 |  |
|  | ≤2/week | 45.00 | 23.08 | 46.00 | 23.59 | 0.51 | 58.00 | | 29.74 | 56.00 | 28.72 | -1.03 |  |
|  | ≥3/week | 50.00 | 25.64 | 48.00 | 24.62 | -1.03 | 51.00 | | 26.15 | 47.00 | 24.10 | -2.05 |  |
|  | Missing | 0.00 | 0.00 | 0.00 | 0.00 | 0.00 | 1.00 | | 0.51 | 0.00 | 0.00 | -0.51 |  |
| Toxic exposure | No | 176.00 | 90.26 | 179.00 | 91.79 | 1.54 | 167.00 | | 85.64 | 169.00 | 86.67 | 1.03 |  |
|  | Yes | 17.00 | 8.72 | 15.00 | 7.69 | -1.03 | 28.00 | | 14.36 | 26.00 | 13.33 | -1.03 |  |
|  | Missing | 2.00 | 1.03 | 1.00 | 0.51 | -0.51 | 0.00 | | 0.00 | 0.00 | 0.00 | 0.00 |  |
| Vegetables | ≤2/week | 26.00 | 13.33 | 24.00 | 12.31 | -1.03 | 27.00 | | 13.85 | 23.00 | 11.79 | -2.05 |  |
|  | 3–4/week | 42.00 | 21.54 | 51.00 | 26.15 | 4.62 | 48.00 | | 24.62 | 49.00 | 25.13 | 0.51 |  |
|  | ≥5/week | 127.00 | 65.13 | 119.00 | 61.03 | -4.10 | 119.00 | | 61.03 | 122.00 | 62.56 | 1.54 |  |
|  | Missing | 0.00 | 0.00 | 1.00 | 0.51 | 0.51 | 1.00 | | 0.51 | 1.00 | 0.51 | 0.00 |  |
| White meat | <1/week | 25.00 | 12.82 | 25.00 | 12.82 | 0.00 | 34.00 | | 17.44 | 45.00 | 23.08 | 5.64 |  |
|  | 1–2/week | 117.00 | 60.00 | 120.00 | 61.54 | 1.54 | 108.00 | | 55.38 | 104.00 | 53.33 | -2.05 |  |
|  | ≥3/week | 53.00 | 27.18 | 50.00 | 25.64 | -1.54 | 52.00 | | 26.67 | 46.00 | 23.59 | -3.08 |  |
|  | Missing | 0.00 | 0.00 | 0.00 | 0.00 | 0.00 | 1.00 | | 0.51 | 0.00 | 0.00 | -0.51 |  |

**Supplemental table 3** – Goodness-of-fit and distance metrics comparing real and synthetic data, stratified by cases and controls

| **stratum** | **predictor** | **TV** | **JS** | **Hellinger** | **p-value chi2** | **Cramer's V** | **pMSE** | **spMSE** |
| --- | --- | --- | --- | --- | --- | --- | --- | --- |
| Controls | Toxic exposure | 0.015 | 0.001 | 0.025 | 0.785 | 0.035 | 0.0003 | 0.484 |
| Controls | Smoke | 0.005 | 0.000 | 0.004 | 0.994 | 0.005 | 0.0000 | 0.012 |
| Controls | Alcohol | 0.010 | 0.000 | 0.022 | 0.832 | 0.031 | 0.0002 | 0.368 |
| Controls | Pasta | 0.031 | 0.001 | 0.026 | 0.776 | 0.036 | 0.0003 | 0.508 |
| Controls | Rice | 0.005 | 0.000 | 0.004 | 0.993 | 0.006 | 0.0000 | 0.014 |
| Controls | Meat | 0.087 | 0.004 | 0.063 | 0.213 | 0.089 | 0.0020 | 3.096 |
| Controls | Raw meat | 0.031 | 0.001 | 0.028 | 0.733 | 0.040 | 0.0004 | 0.622 |
| Controls | White meat | 0.015 | 0.000 | 0.013 | 0.939 | 0.018 | 0.0001 | 0.125 |
| Controls | Fish | 0.021 | 0.004 | 0.072 | 0.350 | 0.073 | 0.0013 | 2.098 |
| Controls | Vegetables | 0.051 | 0.003 | 0.064 | 0.530 | 0.075 | 0.0014 | 2.211 |
| Controls | Fruit | 0.041 | 0.002 | 0.039 | 0.557 | 0.055 | 0.0008 | 1.171 |
| Controls | Cheese | 0.026 | 0.002 | 0.043 | 0.706 | 0.060 | 0.0009 | 1.398 |
| Controls | Eggs | 0.026 | 0.000 | 0.022 | 0.829 | 0.031 | 0.0002 | 0.374 |
| Controls | Sweets | 0.041 | 0.001 | 0.034 | 0.641 | 0.048 | 0.0006 | 0.890 |
| Controls | Coffee | 0.026 | 0.000 | 0.022 | 0.946 | 0.031 | 0.0002 | 0.373 |
| Controls | Tea | 0.010 | 0.000 | 0.009 | 0.972 | 0.012 | 0.0000 | 0.057 |
| Controls | Milk | 0.026 | 0.000 | 0.020 | 0.852 | 0.029 | 0.0002 | 0.320 |
| Controls | Soft drinks | 0.046 | 0.002 | 0.048 | 0.625 | 0.067 | 0.0011 | 1.755 |
| Cases | Toxic exposure | 0.010 | 0.000 | 0.011 | 0.769 | 0.015 | 0.0001 | 0.086 |
| Cases | Smoke | 0.000 | 0.000 | 0.000 | 1.000 | 0.000 | 0.0000 | 0.000 |
| Cases | Alcohol | 0.036 | 0.001 | 0.028 | 0.441 | 0.039 | 0.0004 | 0.593 |
| Cases | Pasta | 0.036 | 0.002 | 0.057 | 0.683 | 0.062 | 0.0010 | 1.495 |
| Cases | Rice | 0.026 | 0.000 | 0.021 | 0.948 | 0.030 | 0.0002 | 0.360 |
| Cases | Meat | 0.062 | 0.006 | 0.083 | 0.223 | 0.106 | 0.0028 | 4.377 |
| Cases | Raw meat | 0.031 | 0.002 | 0.055 | 0.724 | 0.058 | 0.0008 | 1.321 |
| Cases | White meat | 0.056 | 0.004 | 0.071 | 0.396 | 0.087 | 0.0019 | 2.974 |
| Cases | Fish | 0.036 | 0.002 | 0.056 | 0.479 | 0.061 | 0.0009 | 1.473 |
| Cases | Vegetables | 0.021 | 0.000 | 0.022 | 0.947 | 0.031 | 0.0002 | 0.368 |
| Cases | Fruit | 0.041 | 0.001 | 0.032 | 0.849 | 0.045 | 0.0005 | 0.803 |
| Cases | Cheese | 0.067 | 0.005 | 0.076 | 0.315 | 0.095 | 0.0023 | 3.545 |
| Cases | Eggs | 0.046 | 0.004 | 0.061 | 0.420 | 0.085 | 0.0018 | 2.819 |
| Cases | Sweets | 0.051 | 0.003 | 0.065 | 0.518 | 0.076 | 0.0015 | 2.269 |
| Cases | Coffee | 0.041 | 0.003 | 0.064 | 0.536 | 0.075 | 0.0014 | 2.179 |
| Cases | Tea | 0.036 | 0.002 | 0.056 | 0.688 | 0.062 | 0.0009 | 1.475 |
| Cases | Milk | 0.026 | 0.001 | 0.029 | 0.887 | 0.041 | 0.0004 | 0.642 |
| Cases | Soft drinks | 0.021 | 0.002 | 0.054 | 0.726 | 0.058 | 0.0008 | 1.313 |

**Supplemental Table 4** – Results from univariate logistic regression analysis in real and synthetic database

| **predictor** | **level** | **OR (95% CI) - Real** | **OR (95% CI) - Synthetic** | **p-value - Real** | **p-value - Synthetic** |
| --- | --- | --- | --- | --- | --- |
| Alcohol | Yes | 0.81 (0.53–1.25) | 0.93 (0.61–1.41) | 0.344 | 0.720 |
| Cheese | 1–2/week | 0.96 (0.55–1.68) | 1.24 (0.69–2.26) | 0.896 | 0.473 |
|  | ≥3/week | 0.78 (0.46–1.32) | 1.00 (0.56–1.78) | 0.352 | 0.996 |
| Coffee | ≤4/week | 1.19 (0.52–2.68) | 0.94 (0.43–2.03) | 0.681 | 0.873 |
|  | ≥5/week | 0.76 (0.42–1.38) | 0.65 (0.37–1.14) | 0.360 | 0.134 |
| Eggs | 1–2/week | 0.95 (0.62–1.46) | 0.76 (0.50–1.16) | 0.816 | 0.205 |
|  | ≥3/week | 1.36 (0.58–3.20) | 0.75 (0.27–2.07) | 0.475 | 0.583 |
| Fish | ≥1/week | 0.53 (0.34–0.83) | 0.43 (0.28–0.67) | 0.005 | 0.003 |
| Fruit | 3–4/week | 1.36 (0.65–2.88) | 1.73 (0.81–3.68) | 0.416 | 0.156 |
|  | ≥5/week | 0.94 (0.54–1.64) | 1.54 (0.89–2.67) | 0.819 | 0.127 |
| Meat | 1–2/week | 0.73 (0.35–1.52) | 0.41 (0.21–0.79) | 0.406 | 0.008 |
|  | ≥3/week | 0.72 (0.35–1.49) | 0.52 (0.26–1.03) | 0.373 | 0.059 |
| Milk | ≤4/week | 1.38 (0.82–2.32) | 1.65 (0.98–2.78) | 0.221 | 0.058 |
|  | ≥5/week | 1.60 (1.01–2.53) | 1.46 (0.92–2.30) | 0.044 | 0.108 |
| Pasta | 3–4/week | 0.58 (0.35–0.95) | 0.61 (0.38–1.00) | 0.031 | 0.050 |
|  | ≥5/week | 0.64 (0.38–1.09) | 0.79 (0.48–1.32) | 0.099 | 0.376 |
| Raw meat | <1/week | 1.45 (0.86–2.47) | 1.79 (1.04–3.08) | 0.167 | 0.035 |
|  | ≥1/week | 1.23 (0.77–1.98) | 1.22 (0.77–1.94) | 0.382 | 0.391 |
| Rice | 1–2/week | 1.01 (0.64–1.59) | 0.93 (0.60–1.46) | 0.965 | 0.761 |
|  | ≥3/week | 0.47 (0.24–0.93) | 0.39 (0.20–0.79) | 0.029 | 0.008 |
| Smoke | Yes | 1.38 (0.91–2.08) | 1.34 (0.89–2.03) | 0.132 | 0.162 |
| Soft drinks | ≤2/week | 1.25 (0.78–1.98) | 0.96 (0.61–1.53) | 0.354 | 0.876 |
|  | ≥3/week | 1.56 (0.87–2.80) | 1.70 (0.95–3.05) | 0.140 | 0.075 |
| Sweets | 1–2/week | 0.87 (0.53–1.44) | 1.48 (0.89–2.45) | 0.595 | 0.130 |
|  | ≥3/week | 1.07 (0.65–1.76) | 1.45 (0.88–2.38) | 0.788 | 0.146 |
| Tea | ≤2/week | 1.52 (0.93–2.46) | 1.34 (0.83–2.16) | 0.092 | 0.238 |
|  | ≥3/week | 1.20 (0.74–1.95) | 1.07 (0.66–1.76) | 0.462 | 0.773 |
| Toxic exposure | Yes | 1.74 (0.92–3.29) | 1.84 (0.94–3.59) | 0.091 | 0.075 |
| Vegetables | 3–4/week | 1.10 (0.56–2.17) | 1.00 (0.50–2.01) | 0.782 | 0.994 |
|  | ≥5/week | 0.90 (0.50–1.63) | 1.07 (0.57–2.00) | 0.734 | 0.833 |
| White meat | 1–2/week | 0.68 (0.38–1.21) | 0.48 (0.28–0.84) | 0.189 | 0.010 |
|  | ≥3/week | 0.72 (0.38–1.37) | 0.51 (0.27–0.96) | 0.319 | 0.037 |
